# Supplementary material for: Nonlinear optical observation of coherent acoustic Dirac plasmons in thin-film topological insulators
Source: Nat Commun. 2016 Sep 30;7:13054. doi: 10.1038/ncomms13054 (PMC5056522; doi:10.1038/ncomms13054)
Supplement: Supplementary Information — Supplementary Figures 1-3. [file ncomms13054-s1.pdf]

Nonlinear Optical Observation of Coherent Acoustic Dirac Plasmons in Thin-Film Topological Insulators

Supplementary Information

Supplementary Figures

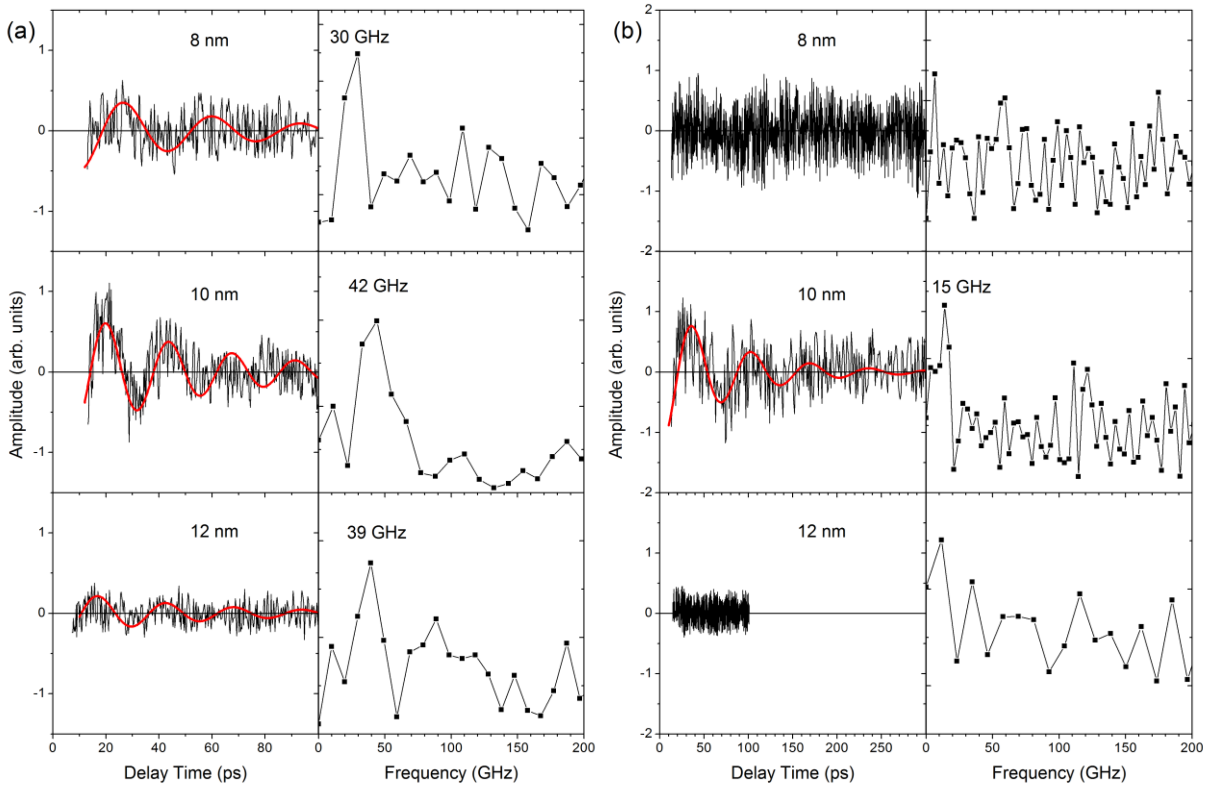

Supplementary Figure 1 | An analyses of the oscillatory part of the TSHG traces. The extracted oscillatory part of the TSHG traces (left columns) and their fast Fourier transforms (FFT) (right columns) for Bi_2Se_3 films of various thicknesses as indicated in nm, which were measured in the P_{in} - S_{pump} - P_{out} (a) and S_{in} - P_{pump} - S_{out} (b) polarization geometries. Figure (a) clearly demonstrates ~42 GHz oscillations measured in the P_{in} - S_{pump} - P_{out} polarization geometry for the 10 nm thick film, which however become significantly suppressed (although still observable) and show slightly decreased frequencies for thinner and thicker films. Alternatively, ~15 GHz oscillations which were measured in the S_{in} - P_{pump} - S_{out} light polarization geometry for the 10 nm thick film are completely damped for thinner and thicker films (the corresponding FFT curves show just a noise level) [Figure (b)]. Red curves present the result of the fit to the damped cosine function using FFT frequencies and damping coefficients 50 ps (a) and 80 ps (b).

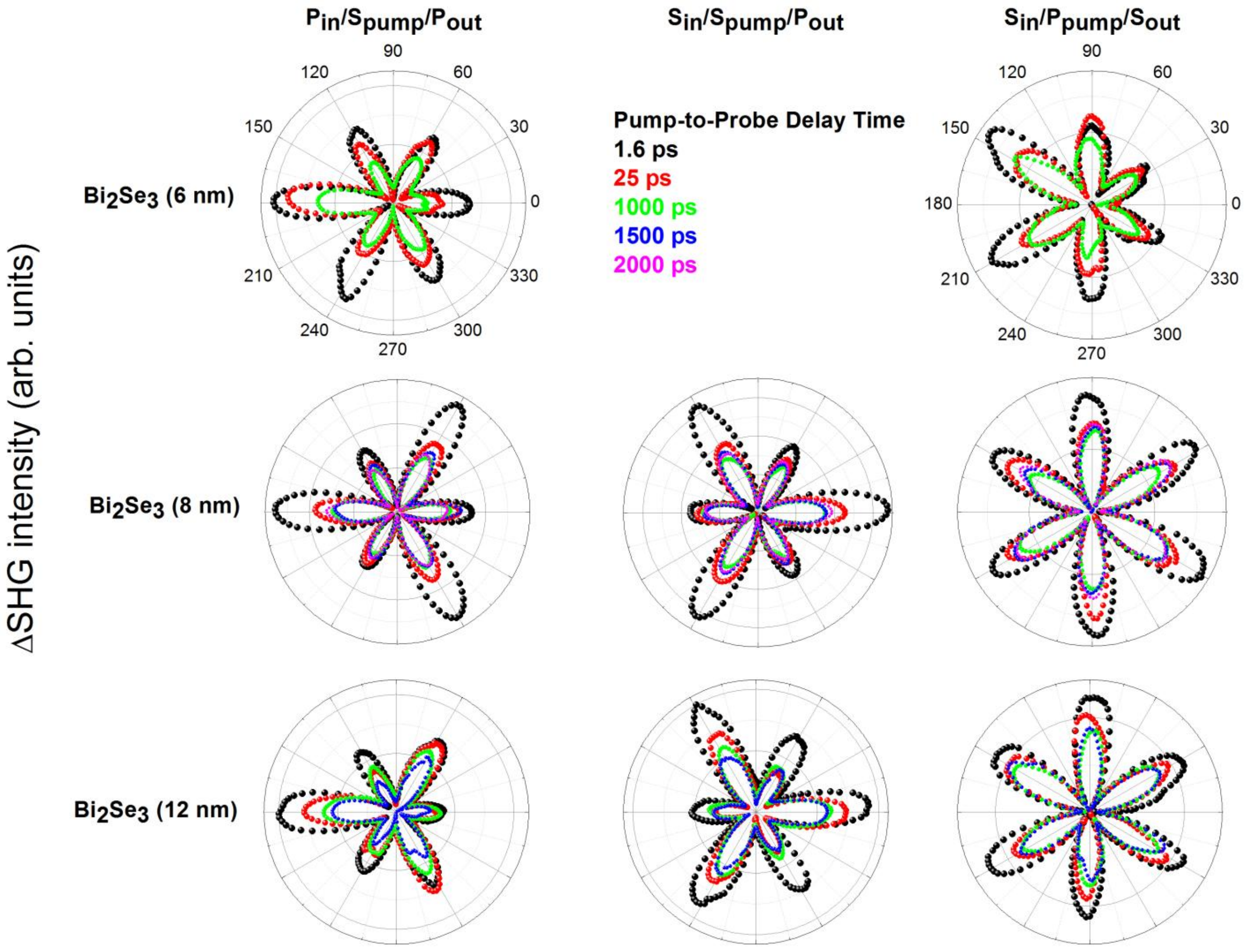

Supplementary Figure 2 | TSHGRA patterns for Bi_2Se_3 films of various thicknesses. The TSHGRA patterns of the 6, 8, and 12 nm thick Bi_2Se_3 films (rows) measured in the P_{in} - S_{pump} - P_{out}, S_{in} - S_{pump} - P_{out}, and S_{in} - P_{pump} - S_{out} light polarization geometries (columns) at different pump-to-probe delay times as indicated by the corresponding colors. The TSHGRA patterns of the 6 nm thick film reveal a significant distortion of the rotational symmetry in a similar way as that observed for the stationary SHG response [Ref. 52 in the paper]. The TSHGRA patterns measured in the S_{in} - P_{pump} - S_{out} light polarization geometry do not show any temporal changes in rotational symmetry for the films of various thicknesses, similar to the experimental findings observed for the 10 nm thick film (see figure 2 in the original paper). In contrast, the threefold-to-sixfold symmetry change of the TSHGRA patterns can be observed in the P_{in} -

S_{pump} - P_{out} and S_{in} - S_{pump} - P_{out} light polarization geometries. The largest effect is for the 10 nm thick film (see figure 1 in the original paper), smaller for the 8 nm thick film, and is significantly suppressed for the films with d <8 nm and d >10 nm.

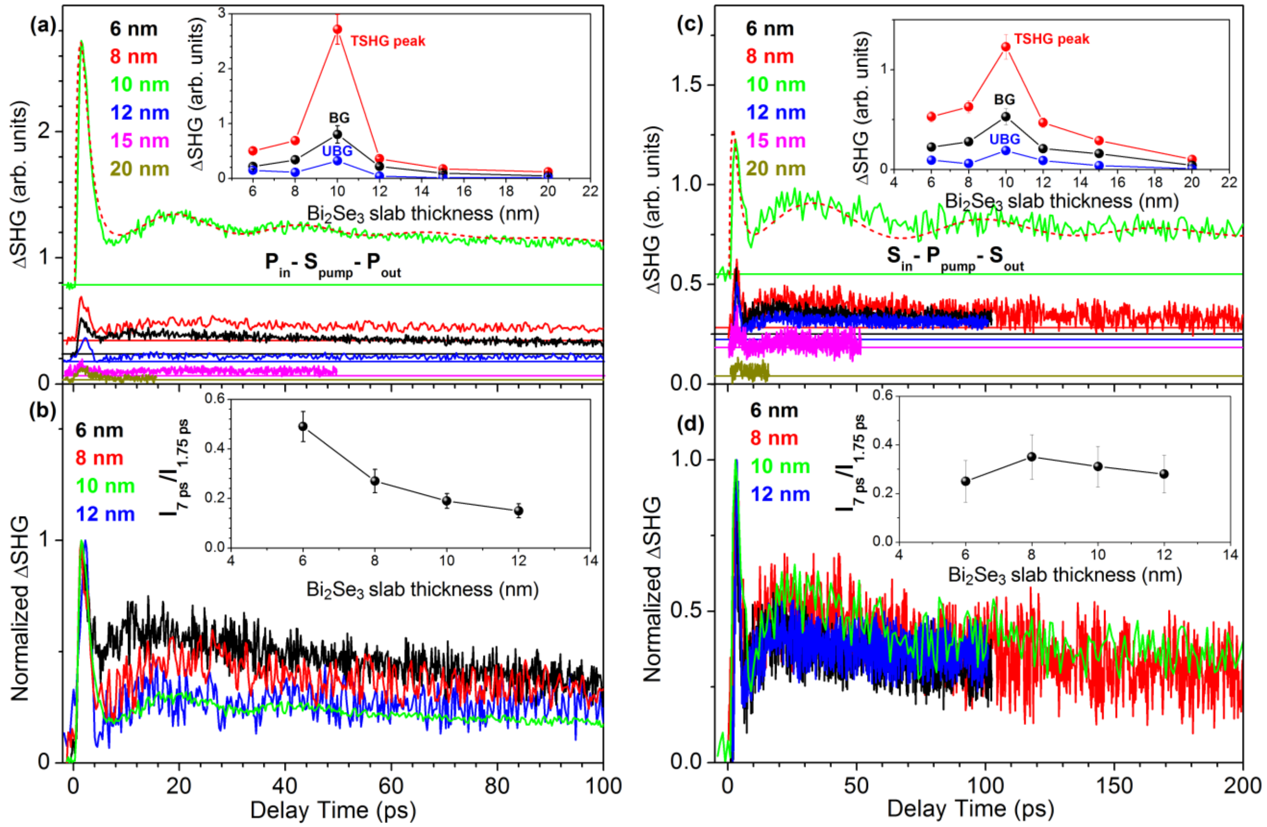

Supplementary Figure 3 | TSHG traces for Bi_2Se_3 films of various thicknesses. The TSHG traces of Bi_2Se_3 films of various thicknesses as indicated by the corresponding colors measured in the P_{in} - S_{pump} - P_{out} [(a) and (b)] and S_{in} - P_{pump} - S_{out} [(c) and (d)] polarization geometries. Figures (b) and (d) show the same curves shown in (a) and (c), but being normalized. The TSHG traces shown in (a) and (c) for the 10 nm thick films and red dotted curves presenting the result of the fit are the same as those shown in figures 1 and 2 of the original paper. Insets in (a) and (c) present the d dependences of the TSHG-peak, TSHG-BG, and TSHG-UBG intensities. Insets in (b) and (d) show the d dependences of the ratio of the intensities at ~7 ps (a dip of the TSHG traces) to that at ~1.7 ps (TSHG-peak). One can clearly see that the oscillatory part is superimposed on the intensity of the second peak of the TSHG response. Consequently, the oscillatory part intensity is significantly suppressed for the films with d <8 nm and d >10 nm, despite the second peak of the TSHG response is always present.
